# Supplementary material for: Multiarm, non-randomised, single-centre feasibility study—investigation of the differential biology between benign and malignant renal masses using advanced magnetic resonance imaging techniques (IBM-Renal): protocol
Source: BMJ Open. 2024 Oct 26;14(10):e083980. doi: 10.1136/bmjopen-2024-083980 (PMC11529771; doi:10.1136/bmjopen-2024-083980)
Supplement: Supplementary file 1 [file bmjopen-2024-083980.reviewer_comments.pdf]

PEER REVIEW HISTORY

BMJ Open publishes all reviews undertaken for accepted manuscripts. Reviewers are asked to complete a checklist review form and are provided with free text boxes to elaborate on their assessment. These free text comments are reproduced below.

ARTICLE DETAILS

Title (Provisional)

Protocol for a multi-arm, non-randomised, single-centre feasibility study: Investigation of the differential biology between benign and malignant renal masses using advanced magnetic resonance imaging techniques (IBM-Renal)

Authors

Horvat-Menih, Ines; McLean, Mary A; Zamora-Morales, Maria Jesus; Wylot, Marta; Kaggie, Joshua; Khan, Alixander S; Gill, Andrew B; Duarte, Joao; Locke, Matthew J; Mendichovszky, Iosif; Li, Hao; Priest, Andrew N; Warren, Anne Y; Welsh, Sarah J; Jones, James O; Armitage, James N; Mitchell, Thomas J; Stewart, Grant D; Gallagher, FA

VERSION 1 - REVIEW

**Reviewer**	**1**
**Name**	**Anari, Pouria Yazdian**
**Affiliation**	**National Institutes of Health**
**Date**	**08-Apr-2024**
**COI**	**None**

The protocol for the study "Investigation of the differential biology between benign and malignant renal masses using advanced magnetic resonance imaging techniques (IBM-Renal): a multi-arm, non-randomised feasibility study" presents a detailed and structured research plan aimed at addressing a critical gap in the clinical management of renal masses. Below are some suggestions to potentially enhance the study protocol:

1. Patient-Reported Outcomes: Adding patient-reported outcome measures (PROMs) related to quality of life, symptom burden, and psychological impact of undergoing advanced MRI scans for renal mass characterization could offer insights into the patient experience.

2. Economic Analysis: Incorporating a health economic analysis component to evaluate the cost-effectiveness of employing these MRI techniques in the clinical management of renal masses could provide compelling evidence.

These are just some suggestions and are not necessary to add to the protocol.

**Reviewer**	**2**
**Name**	**Warren, Hannah**
**Affiliation**	**University College London Division of Surgery and Interventional Science**
**Date**	**26-Apr-2024**
**COI**	**None**

Overarching comments:

Management of early-stage renal tumours is becoming increasingly nuanced as we have a better understanding of the natural history of small renal masses, (some) diagnostic tools, and a range of management options (surveillance, ablation, surgery). The authors are commendably investigating new non-invasive modalities to help risk stratify small renal tumours stating 'The ultimate goal is to provide non-invasive tools for early detection of small aggressive renal tumours to enable timely surgical intervention'. We know from observational studies e.g. DISSRM registry that the majority of small renal masses (approx. 80%) remain stable or regress over several years of follow up. Only the minority progress, and we currently lack tools to reliably risk-stratify patients leading to both under and over treatment. Therefore the 'ultimate goal' the authors outline would address an important unmet clinical need.

I noted that there was some inconsistency in the terminology in the aims and objective of the study. Benign v malignant is not equivalent to aggressive v non aggressive. It is a good starting point to spare patients with benign tumours invasive treatment, but we would also like to spare patients with indolent, malignant disease from overtreatment knowing that they have an excellent prognosis on surveillance. It should also be commented somewhere that a binary cut off such as benign v malignant is perhaps overly simplistic, and renal tumours actually fall on a spectrum of aggressiveness. Further, in some patients there will be a higher threshold to consider active treatment, for example if they have significant comorbidities.

No note was made of different histological subtypes within renal cell carcinoma, but we know that papillary RCC can have very characteristic appearances on MRI. How will you make sure you include different RCC subtypes in all three arms?

Please read and consider reporting your trial according to the SPIRIT-Path extension in addition to SPIRIT checklist.

If the participant agrees to have an optional second scan of another cohort within 14 days, how will the scan type be determined?

The study is described interchangeably as a pilot and feasibility trial, yet these are not the same thing. Please see https://www.journalslibrary.nihr.ac.uk/information-for-authors/Full-Final-Report/report-contents/report-types/pilot-and-feasibility-studies.htm

The description of study methods is somewhat lacking. Please clearly define the primary objective of this feasibility/pilot(?) trial and how it will be assessed. In the abstract you state the primary objective is to investigate whether novel MRI techniques can identify the differences between benign and malignant tumours, but that is actually the objective of the follow-on work, rather than the pilot/feasibility protocol you are reporting here. In the full text a stated objective is to ‘acquire preliminary data to optimise future imaging protocols’. How will this be done? Will you test different protocols? How will protocols be optimised? If this is your primary outcome your description of the methods for this must be more robust. For exploratory outcomes e.g. description of lactate-to-pyruvate ratio, will you attempt to define a threshold that identifies benign v malignant lesions? Please review this section.

Other comments:

In the opening sentence of the introduction you state the age-standardised incidence of kidney cancer in the UK is 10.2 per 100,000 and reference Capitano's European Urology 2019 paper on epidemiology of renal cancer. However, in the cited reference the age-standardised rate is reported as 8.2 per 100,000 for the UK. Please review and clarify.

In the second sentence you state that RCCs ‘exhibit a variable degree of aggressiveness depending on the histology’. Firstly, the cited reference discusses the role of molecular imaging in renal cancer, rather than oncological outcomes of different tumour histotypes, so I do not think it is an appropriate reference here. Secondly, it is a little simplistic. While we may use histological subtype clinically as a surrogate for aggressiveness, as it can give us an idea of prognosis, there are other tumour- and patient-related factors at play, as well as complex immune-mediated interactions between the two. Please revise this sentence appropriately.

In the third sentence you state ‘Management options range from active surveillance through to radical surgical resection depending on the tumour type (3)’. This is incorrect. The tumour type is only known in the minority of cases pre-operatively as biopsy is not widely used. The cited international guidelines do not dictate the management option most appropriate for each tumour type. In theory, management options are considered based on tumour and patient-related factors (taking into account tumour location, complexity, patient comorbidities, surgical history, renal function etc) and a shared decision made with the patient.

First page, line 20 change ‘increased detection and improved management’ to ‘earlier detection and treatment’

First page, line 42/43 suggest changing the wording to ‘and therefore help risk-stratify tumours to inform management decisions’.

You say you ‘calculated’ a sample size of 30, but a description of the calculation is not provided. Did you actually calculate the sample size using a statistical method or just choose a pragmatic number for a priming pilot study? Please make this clear.

In methods, please state the time interval between injection or drink and the scan.

Page 12, line 21 students -> studies?

Format of reference 3 is incorrect.3. Professionals SO. Uroweb. [cited 2021 Jun 4]. EAU Guidelines: Renal Cell Carcinoma. Available from: https://uroweb.org/guideline/renal-cell-carcinoma/

doi: 10.1016/j.eururo.2023.07.013 is a more up to date systematic review on this topic than ref 24 (Wilson et al)

**Reviewer**	**3**
**Name**	**Hesketh, Richard**
**Affiliation**	**University College London, Centre of Medical Imaging**
**Date**	**09-May-2024**
**COI**	**None.**

An interesting and important feasibility and initial proof of concept study.

A couple of minor comments/clarifications are attached.

Page 9, line 29. Slight discrepancy between timepoint 4 and the flowchart. Please clarify whether patients with patients will **only** have a research biopsy at a standard of care biopsy appointment? If the majority of oncocytomas are recruited from the retrospective cohort having already had a SoC biopsy you won’t have research tissue from these patients.

Page 11, Table 2. Please clarify the schedule of repeated blood sampling at each visit - currently my understanding from (c) is mandatory blood sampling at every visit for biochemical series, LFTs and possibly other tests but as per (d) FBC only included in this if not performed <14d?

VERSION 1 - AUTHOR RESPONSE

Reviewer #1:

The protocol for the study "Investigation of the differential biology between benign and malignant renal masses using advanced magnetic resonance imaging techniques (IBM-Renal): a multi-arm, non-randomised feasibility study" presents a detailed and structured

research plan aimed at addressing a critical gap in the clinical management of renal masses. Below are some suggestions to potentially enhance the study protocol:

- Patient-Reported Outcomes: Adding patient-reported outcome measures (PROMs) related to quality of life, symptom burden, and psychological impact of undergoing advanced MRI scans for renal mass characterization could offer insights into the patient experience.
- Economic Analysis: Incorporating a health economic analysis component to evaluate the cost-effectiveness of employing these MRI techniques in the clinical management of renal masses could provide compelling evidence.

These are just some suggestions and are not necessary to add to the protocol.

Thank you for your encouraging review! Both suggestions are of great interest to us, and will be considered for the large-scale study, which will be based on the preliminary data coming from the IBM-renal.

Reviewer #2:

Overarching comments:

Management of early-stage renal tumours is becoming increasingly nuanced as we have a better understanding of the natural history of small renal masses, (some) diagnostic tools, and a range of management options (surveillance, ablation, surgery). The authors are commendably investigating new non-invasive modalities to help risk stratify small renal tumours stating ‘The ultimate goal is to provide non-invasive tools for early detection of small aggressive renal tumours to enable timely surgical intervention’. We know from observational studies e.g. DISSRM registry that the majority of small renal masses (approx. 80%) remain stable or regress over several years of follow up. Only the minority progress, and we currently lack tools to reliably risk-stratify patients leading to both under and over treatment. Therefore the ‘ultimate goal’ the authors outline would address an important unmet clinical need.

Thank you for this encouraging comment! Indeed, we agree that this is an important unmet clinical need which requires addressing.

I noted that there was some inconsistency in the terminology in the aims and objective of the study. Benign v malignant is not equivalent to aggressive v non-aggressive. It is a good starting point to spare patients with benign tumours invasive treatment, but we would also like to spare patients with indolent, malignant disease from overtreatment knowing that they have an excellent prognosis on surveillance. It should also be commented somewhere that a binary cut off such as benign v malignant is perhaps overly simplistic, and renal tumours actually fall on a spectrum of aggressiveness. Further, in some patients there will be a higher threshold to consider active treatment, for example if they have significant comorbidities.

Thank you, this is a very good point, and fully agreed with. We have made changes accordingly, please see:

Page 3:

"An important unmet clinical need is that conventional imaging methods cannot reliably distinguish aggressive RCC subtypes from **indolent** renal masses (3)."

"Therefore, it is not clear to what extent the ratio between glycolytic and oxidative metabolism varies **across aggressiveness** of renal neoplasms (17,19)."

"The question to be addressed in this study is whether MRI can be used to **phenotype the aggressiveness of renal masses**, and can therefore implemented to help risk-stratify tumours to inform management decisions."

No note was made of different histological subtypes within renal cell carcinoma, but we know that papillary RCC can have very characteristic appearances on MRI. How will you make sure you include different RCC subtypes in all three arms?

Thank you, we are we are indeed aware of this but have kept the description of the protocol general as we are interested to phenotype any RCC histological subtype without specific allocation to the arms, and compare those to the imaging features of the oncocytoma (and "Oncocytic renal cell neoplasm, favouring an oncocytoma" as determined on biopsy) and learn from this as it is a feasibility study.

Please read and consider reporting your trial according to the SPIRIT-Path extension in addition to SPIRIT checklist.

Thank you for this suggestion – we have expanded the SPIRIT checklist with SPIRIT-Path extension.

If the participant agrees to have an optional second scan of another cohort within 14 days, how will the scan type be determined?

Thank you for referring to this, we have updated accordingly based on our study protocol, please see Page 6 under *Participant selection*:

"The recruited participant will be allocated to one of these imaging techniques by the Chief Investigator. This will be based on the availability of kits for HP ^{13}C-MRI, availability of the deuterated glucose drink for the DMI study and/or availability of the research MRI scanner."

And on Page 7 under *Interventions*:

"3. For those not taking part in Part 2 above, an optional scan with another imaging technique within 14 days of the first scan. The second imaging arm will be determined as detailed above; that is by the Chief Investigator based on the availability of the imaging kits and the research MRI scanner."

The study is described interchangeably as a pilot and feasibility trial, yet these are not the same thing. Please see:
https://www.journalslibrary.nihr.ac.uk/information-for-authors/Full-Final-Report/report-contents/report-types/pilot-and-feasibility-studies.htm

Thank you for noticing this – indeed it is a feasibility study, we have now changed accordingly on Page 4:

"This **feasibility study** will explore the role of multimodal MRI in characterising localised renal masses and how it can exploit the known biological differences between benign and malignant lesions."

The description of study methods is somewhat lacking. Please clearly define the primary objective of this feasibility/pilot(?) trial and how it will be assessed. In the abstract you state the primary objective is to investigate whether novel MRI techniques can identify the differences between benign and malignant tumours, but that is actually the objective of the follow-on work, rather than the pilot/feasibility protocol you are reporting here. In the full text a stated objective is to 'acquire preliminary data to optimise future imaging protocols'. How will this be done? Will you test different protocols? How will protocols be optimised? If this is your primary outcome your description of the methods for this must be more robust. For exploratory outcomes e.g. description of lactate-to-pyruvate ratio, will you attempt to define a threshold that identifies benign v malignant lesions? Please review this section.

Indeed, we agree that the feasibility aspect of the study should have been stated more clearly, please see our changes on Page 6 under *Study design and objectives*:

"The IBM-Renal (**I**nvestigation of differential biology of **B**enign and **M**alignant **Renal** masses using advanced magnetic resonance imaging techniques) study is designed as a feasibility study to acquire preliminary data **with set imaging protocols as defined based on our previous work, and these results will be used to optimise future imaging protocols and to inform large-scale studies.** The study will be conducted as a non-randomised, physiological imaging study in patients with localised renal masses. The study will be conducted at a single site: Addenbrooke's Hospital, Cambridge University Hospitals NHS Foundation Trust. For all imaging studies, the primary objective will be **the technical development of each imaging technique with ultimate aim to understand whether the methods can identify any differences between benign and malignant tumours**, including measuring the ^{13}C-pyruvate-to-lactate conversion with HP ^{13}C-MRI, quantifying sodium concentration using ^{23}Na-MRI, detecting ^{2}H-glucose and its metabolites using DMI. Secondary objectives will aim to compare the imaging techniques to understand if they can produce complementary information. As an exploratory part of the study, the objective will be to link

the imaging data with clinical data and tissue molecular analyses for biological validation of the novel MRI techniques. **Diagnostic accuracy analysis is not foreseen at this stage.**"

Other comments:

In the opening sentence of the introduction you state the age-standardised incidence of kidney cancer in the UK is 10.2 per 100,000 and reference Capitano's European Urology 2019 paper on epidemiology of renal cancer. However, in the cited reference the age-standardised rate is reported as 8.2 per 100,000 for the UK. Please review and clarify.

Thank you for noticing this mistake, we have corrected accordingly, please see Page 3.

In the second sentence you state that RCCs 'exhibit a variable degree of aggressiveness depending on the histology'. Firstly, the cited reference discusses the role of molecular imaging in renal cancer, rather than oncological outcomes of different tumour histotypes, so I do not think it is an appropriate reference here. Secondly, it is a little simplistic. While we may use histological subtype clinically as a surrogate for aggressiveness, as it can give us an idea of prognosis, there are other tumour- and patient-related factors at play, as well as complex immune-mediated interactions between the two. Please revise this sentence appropriately.

In the third sentence you state 'Management options range from active surveillance through to radical surgical resection depending on the tumour type (3)'. This is incorrect. The tumour type is only known in the minority of cases pre-operatively as biopsy is not widely used. The cited international guidelines do not dictate the management option most appropriate for each tumour type. In theory, management options are considered based on tumour and patient-related factors (taking into account tumour location, complexity, patient comorbidities, surgical history, renal function etc) and a shared decision made with the patient.

Thank you for your suggestions on revising this part of the protocol paper. We have now removed the second and at the same time expanded the third sentence, so that the wording is now:

"Depending on histological subtype and other tumour- and patient related factors (such as tumour location, complexity, patient comorbidities, surgical history, renal function), the management options range from active surveillance to radical surgical resection (2)."

First page, line 20 change 'increased detection and improved management' to 'earlier detection and treatment'.

Changed accordingly.

First page, line 42/43 suggest changing the wording to 'and therefore help risk-stratify tumours to inform management decisions'.

Changed accordingly.

You say you ‘calculated’ a sample size of 30, but a description of the calculation is not provided. Did you actually calculate the sample size using a statistical method or just choose a pragmatic number for a priming pilot study? Please make this clear.

Thank you for this, we have made it clearer with the following change on Page 10:

“Based on the parameters obtained from the HP-^{13}C-MRI we therefore **determined the following sample sizes for priming of the study**: we plan to include up to 30 participants in total: 15 with benign renal masses and 15 with malignant renal masses.”

In methods, please state the time interval between injection or drink and the scan.

We have updated the Methods accordingly, please see Page 9 and 10:

- “HP ^{13}C-MRI: Single intravenous injection of up to 40 mL at 0.4 mL/kg of ^{13}C-pyruvate through an intravenous cannula while the study participant is in position on the scanner bed. **Scanning will begin 12 s after the injection.** There will be an optional repeat injection of ^{13}C-pyruvate at all imaging visits to test for repeatability of the ^{13}C-MRI technique. These will take place within seven days of the first scan.
- ^{23}Na-MRI: Not applicable. No additional research probe will be given to the participant as part of this imaging technique.
- DMI: At each imaging visit the participant will receive a deuterated glucose solution where 60 g of glucose is dissolved in 200 mL of water for injection (WFI) and the dose solution will be adjusted to their body weight at 0.75 g/kg body weight. **Scanning will begin 60 min after the drink ingestion.** There will be an optional repeat deuterated drink at all imaging visits to test for repeatability of the DMI technique. These will take place within seven days of the first scan.”

Page 12, line 21 students -> studies?

Yes thank you, changed accordingly.

Format of reference 3 is incorrect.3. Professionals SO. Uroweb. [cited 2021 Jun 4]. EAU Guidelines: Renal Cell Carcinoma. Available from: https://uroweb.org/guideline/renal-cell-carcinoma/

Changed accordingly.

doi: 10.1016/j.eururo.2023.07.013 is a more up to date systematic review on this topic than ref 24 (Wilson et al)

Agreed, changed accordingly.

Reviewer #3:

An interesting and important feasibility and initial proof of concept study.

A couple of minor comments/clarifications are attached.

Page 9, line 29. Slight discrepancy between timepoint 4 and the flowchart. Please clarify whether patients with patients will only have a research biopsy at a standard of care biopsy appointment? If the majority of oncocytomas are recruited from the retrospective cohort having already had a SoC biopsy you won't have research tissue from these patients.

Thank you for pointing this out, we have now updated the timepoint 4 on Page 7:

4. "An optional research biopsy will be undertaken at standard of care surgery or at biopsy, whichever will be clinically indicated and performed."

Page 11, Table 2. Please clarify the schedule of repeated blood sampling at each visit - currently my understanding from (c) is mandatory blood sampling at every visit for biochemical series, LFTs and possibly other tests but as per (d) FBC only included in this if not performed <14d?

Thank you for pointing this out; we have removed the "full blood count" from the (c) footnote of the Table 2.

VERSION 2 - REVIEW

Reviewer 2

Name Warren, Hannah

Affiliation University College London Division of Surgery and Interventional Science

Date 20-Aug-2024

COI None

Authors have addressed most of my previous comments. The rationale for the sample size is still not clear. As it stands the authors suggest the sample size is powered to detect a difference in median pyruvate-to-lactate conversion constant between tumour types, but there is no description of the calculation, power, alpha error etc. If no calculation has been done, and a pragmatic number has been chosen the authors should just state this otherwise

it is misleading. Further ref 4 still incorrect - please visit link I provided previously for instructions on how to cite this guideline.

VERSION 2 - AUTHOR RESPONSE

Reviewer #2:
Authors have addressed most of my previous comments. The rationale for the sample size is still not clear. As it stands the authors suggest the sample size is powered to detect a difference in median pyruvate-to-lactate conversion constant between tumour types, but there is no description of the calculation, power, alpha error etc. If no calculation has been done, and a pragmatic number has been chosen the authors should just state this otherwise it is misleading.

Thank you for the suggestion to further improve the clarity. We have now changed the wording under “The sample size calculation and outcomes” paragraph on Page 7:
“Based on the parameters obtained from the HP-^{13}C-MRI, pragmatic sample sizes have been determined for priming of the study:…”

Further ref 4 still incorrect - please visit link I provided previously for instructions on how to cite this guideline.
Reference now corrected based on instructions by the EAU Guidelines (https://uroweb.org/eau-guidelines/citing-usage-republication)
Page 12:
4. EAU Guidelines. Edn. presented at the EAU Annual Congress Milan 2023. ISBN 978-94-92671-19-6

If you have selected ‘Yes’ above, please provide details of any competing interests.: None.
We have declared the Competing interests under the Step 5: Authors & Institutions, as well as in the manuscript (Page 17):
“GDS has received educational grants from Pfizer, AstraZeneca and Intuitive Surgical; consultancy fees from Pfizer, MSD, EUSA Pharma and CMR Surgical; Travel expenses from MSD and Pfizer; Speaker fees from Pfizer; Clinical lead (urology) National Kidney Cancer Audit and Topic Advisor for the NICE kidney cancer guideline. S.J.W. is a founder and director of Pinto Medical Consultancy. F.A.G. has research grants from GlaxoSmithKline and AstraZeneca, research support from GE Healthcare, and has consulted for AstraZeneca on behalf of the University of Cambridge.
